# Anion···Anion [AuI_4_]^−^···[AuI_2_]^−^ Complex Trapped in the Solid State by Tetramethylammonium Cations

**DOI:** 10.1021/acs.cgd.2c00749

**Published:** 2022-09-28

**Authors:** Luca Andreo, Rosa M. Gomila, Emanuele Priola, Alessia Giordana, Stefano Pantaleone, Eliano Diana, Ghodrat Mahmoudi, Antonio Frontera

**Affiliations:** † Department of Chemistry, Università degli Studi di Torino, Via Pietro Giuria 7, 10125 Torino, Italy; ‡ Department of Chemistry, Universitat de les Illes Balears, Crta. de Valldemossa km 7.5, 07122 Palma de Mallorca (Baleares), Spain; § Department of Chemistry, Faculty of Science, University of Maragheh, Maragheh 83111-55181, Iran

## Abstract

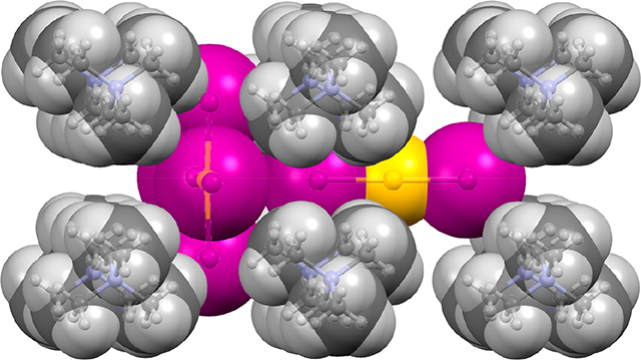

A discrete π-hole···σ-hole dimer is synthesized and X-ray characterized. It presents a perfect thumbtack geometry where the σ-hole of the linear [AuI_2_]^−^ anion points to the π-hole located above the central Au-atom of the [AuI_4_]^−^ anion. Such discrete π-hole···σ-hole dimers are unprecedented in literature, since all mixed-valence gold(I/III) iodide compounds reported to date form infinite ···([AuI_4_]^−^···[AuI_2_]^−^)_*n*_·· chains in the solid state. If an excess of iodine is used for the synthesis, triiodide [I_3_]^−^ ions are partially incorporated into the [AuI_2_]^−^ sites, forming infinite chains. The nature of the anion···anion interaction has been studied considering two possibilities: (i) a π-hole coinage bond or (ii) σ-hole halogen bond using high-level density functional theory calculations, the quantum theory of atoms in molecules, and the noncovalent interaction plot index.

## Abstract 2

A discrete π-hole···σ-hole anion−anion dimer is synthesized and X-ray characterized. It presents a perfect thumbtack geometry where the σ-hole of the linear [AuI_2_]^−^ anion points to the π-hole located above the central Au-atom of the [AuI_4_]^−^ anion. Density functional theory calculations shed light into the nature of the interaction.

## Introduction

The distribution of electron density is anisotropic in covalently bonded atoms, and some of them present σ- or π-hole(s) and σ- or π-lump(s) that may coexist in the outer surface of the same atom.^1^ This phenomenon has been used during the last two decades to rationalize the interactions of all elements of the p-block (groups 13–18).^2^ The adequate comprehension of these interactions has allowed significant achievements in many fields including supramolecular catalysis,^3^ crystal engineering,^4^ host guest chemistry,^5^ medicinal chemistry,^6^ etc. More recently, such approach has been extended to rationalize interactions involving post-transition metals (groups 11^7^ and 12^8^) and also other groups of the d-block of elements. For instance, theoretical and experimental evidence has been recently reported for elements of groups 7^9^ (matere bonds) and 8^10^ (osme bonds).

For group 11 (denoted as coinage or regium bonds),^11^ theoretical and experimental findings have shown that nanoparticles^12^ and halides of Cu, Ag, and Au form attractive interactions with Lewis bases by involving the π-holes (regions of most positive electrostatic potential at their outer surface). Recently, it has been demonstrated that gold in negatively charged species can also function as an acceptor of electron density, establishing coinage bonds (CiBs). In particular, Resnati and collaborators^13^ have shown that [AuCl_4_]^−^ anions are able to act as self-complementary tectons, with the gold and chlorine atoms functioning as CiB donor and acceptor sites, respectively. By using theoretical calculations and crystal engineering, it has been proved that CiBs involving gold(III) centers are strong enough to drive the formation of attractive anion···anion^13^ and anion···neutral nucleophile^14^ interactions, determining the crystal packing of Au(III) derivatives.

**Figure 1:**
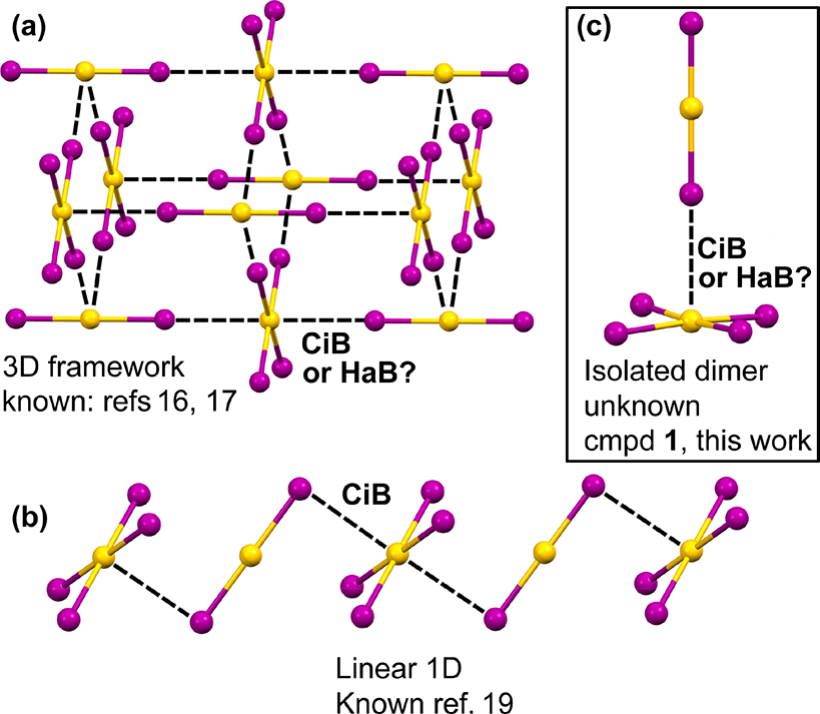
Partial views (cations omitted for clarity) of X-ray structures forming 3D networks (a), linear 1D assemblies (b), and an isolated dimer (c).

Compared to tetrachloro-gold(III) compounds, tetraiodo-gold(III) structures are rare. In fact, only 16 different X-ray structures of tetraiodo-gold(III) are present in the CSD database (see SI for the full list). Most of the investigation on tetraiodo-gold(III) is focused on the synthesis and characterization of organic–inorganic hybrid gold halide perovskites of formulas C_2_[Au^II^_2_][Au^III^_4_] where C is an organic ammonium cation.^15−19^ Multiple anion···anion interactions are commonly formed in these hybrid gold perovskites, as illustrated in Figure 1a, generating 3D frameworks where the diodo-gold(I) and tetraiodo-gold(III) anions are in close contact. The directionality of these contacts, where the σ-hole of the iodine atom in [AuI_2_]^−^ anion points to the π-hole of the [AuI_4_]^−^ anion, complicates the definition of this interaction in terms of donor–acceptor (HaB or CiB). A hybrid structure that forms the 1D assembly depicted in Figure 1b has been also reported, which was described by the original authors as a gold(I/III) iodide chain of face-shared octahedra.^19^ In this particular case, the belt of iodine is interacting with the π-hole of the [AuI_4_]^−^ anion, thus suggesting that this Au(III)···I contact is a CiB.

All previously reported investigations on organic–inorganic hybrid gold halide perovskites used nonsymmetric ammonium cations. We envisaged that the utilization of the small and pseudospherical tetramethylammonium cation would allow the isolation of a sequestered π-hole CiB complex due to the higher capacity of the small cation to solvate the [Au^I^I_2_]···[Au^III^I_4_] and impede the formation of the 3D or 1D supramolecular polymers based on CiBs/HaBs.

## Results and Discussion

**Figure 2:**
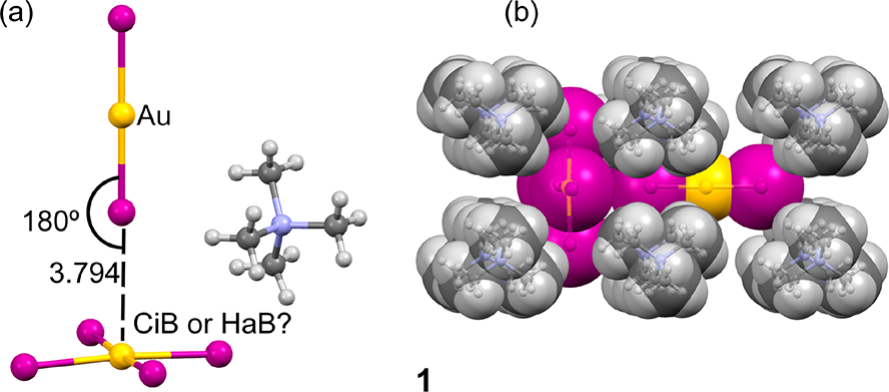
(a) X-ray structure of the asymmetric unit of compound **1**. Distances in Å. (b) CPK representation of the [AuI_2_]^−^···[AuI_4_]^−^ dimer in **1** surrounded by 12 TMA cations.

**Figure 3:**
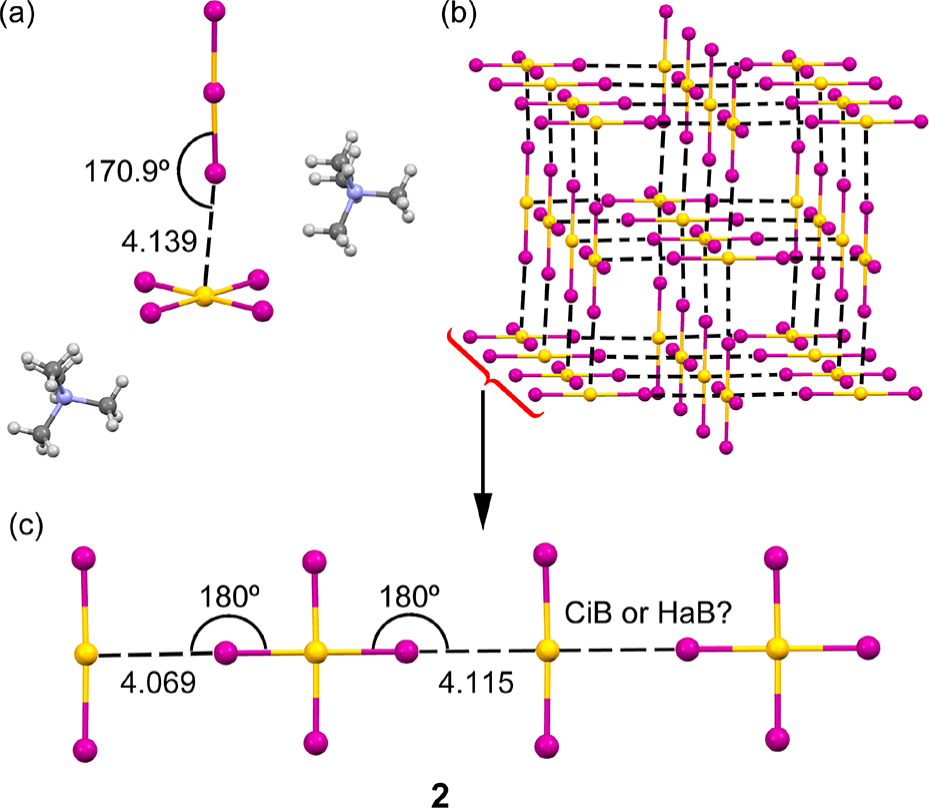
(a) X-ray structure of the asymmetric unit of compound **2**. (b) 3D network observed in the solid state of **2** with indication of the Au(III)···I contacts as dashed lines. (c) Infinite 1D assembly propagated by Au(I)···I contacts. Distances in Å.

The X-ray structure of compounds (Me_4_N)_2_(AuI_2_)(AuI_4_) (**1**) and (Me_4_N)(AuI_2_)_0.5_(AuI_4_)(I_3_)_0.5_ (**2**) are represented in Figures 2 and 3 (see SI for the synthesis and spectroscopic characterization). The site occupancy of the Au atom of [AuI_2_]^−^ in compound **2** is 0.5. In fact, the incorporation of I_3_^–^ into some of the [AuI_2_]^−^ sites is quite common in hybrid C_2_[Au^I^I_2_][Au^III^I_4_] (C = cation) compounds because [AuI_2_]^−^ and I_3_^–^ ions are linear monoanions with similar size.^18^ In contrast, the spectroscopic data and X-ray analysis of **1** are consistent with the chemical formula (Me_4_N)_2_(AuI_2_)(AuI_4_), indicating that I_3_^–^ was not incorporated into the crystal lattice (see SI).

In the crystal packing, both anionic units of **1** are connected through an Au···I interactions where the AuI_4_^–^ anion adopts the usual square-planar conformation and the [AuI_2_] ^–^ anion gets close to the Au(III) center, orthogonal to the AuI_4_^–^ plane. The I–Au···I angles range 88–92°, thus enabling rationalization of the Au···I interactions either as π-hole CiBs^13^ or σ-hole HaBs^2^ since the Au(I)–I···Au(III) angle is perfectly linear (180°) and the Au(III)···I separation is 3.794 Å, that is considerably shorter than the sum of Batsanov’s^20^ van der Waals radii [∑*R*_vdW_(Au + I) = 4.2 Å] and slightly longer than Bondi’s ∑*R*_vdW_ (3.64 Å). It is well-known that Bondi’s^21^*R*_vdW_ values for coinage elements are largely underestimated; therefore, Batsanov’s values are used in this work. In compound **2**, where the site occupancy of the Au(I) atom is 0.5, the Au(III)···I distance is much longer and the directionality of the Au(I)–I···Au(III) interaction is worse (see Figure 3), thus suggesting that the structure directing role of the Au···I contact in **1** is stronger. In fact in compound **2**, a different binding mode is equally dominant in the solid state, where the Au(III)–I bond points to the central Au(I) of the [AuI_2_]^−^ anion (see Figure 3c) with a perfectly linear approximation (180°) and governing the formation of infinite 1D assemblies. A differentiating feature of compound **1** with respect to compound **2** and all previously published C_2_[Au^I^I_2_][Au^III^I_4_] compounds is that the anion···anion complex is isolated and trapped between 12 TMA cations, as can be observed in Figure 2b. That is, only one type of Au···I contact exists in compound **1** that involves the square planar Au(III) metal center. In contrast, the anionic moieties in **2** form a 3D framework, see Figure 3b, bearing the cations as appended residues (see Figure S1 in SI for a representation of both anions and cations) combining Au(III)···I and Au(I)···I interactions.

**Figure 4:**
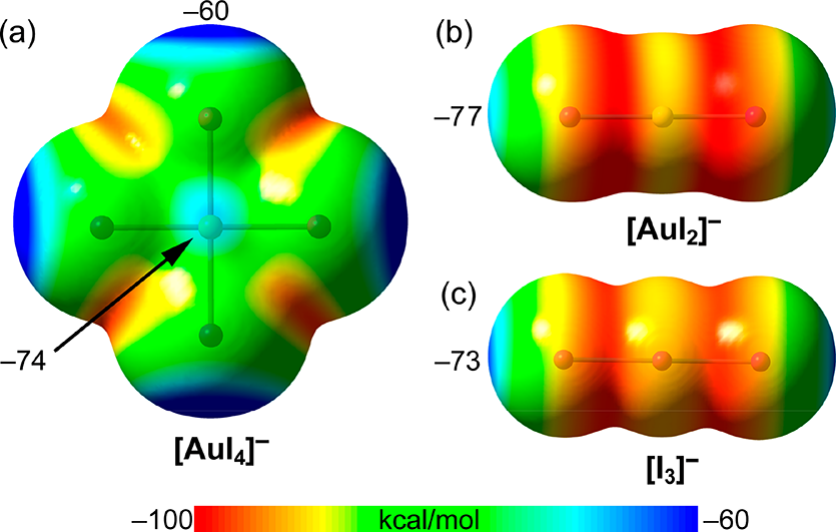
MEP surfaces of [AuI_4_]^−^ (a), [AuI_2_]^−^ (b), and [I_3_]^−^ (c) at the PBE0-D3/def2-TZVPP level of theory (isosurface 0.001 au). The energies at selected points are given in kcal·mol^–1^.

It has been recently proved that the gold center in the [AuCl_4_]^−^ anion can act as an electrophile both experimentally and theoretically.^13^ The short Au(III)···I experimental distance observed in compound **1** (0.5 Å shorter than ∑*R*_vdW_) and the similar theoretical distance obtained for the optimized dimer (3.794 Å, *vide infra*) strongly suggest that the Au(III)···I contact does not simply originate from packing effects. Instead, it suggests that either the negatively charged [AuI_4_]^−^ molecular entity acts as π-hole donor (as described for [AuCl_4_]^−^) or that the negatively charged [AuI_2_]^−^ molecular entity acts as σ-hole donor. To shed some light into this issue and the Au(I)···I interactions contacts of compound **2**, the theoretical study is initially focused on analyzing the molecular electrostatic potential (MEP) surfaces of all anions. The MEP results are summarized in Table 1 and Figure 4, evidencing the expected anisotropy in the MEP distribution of some atoms.

**Table 1:** MEP Values (Vs, in kcal·mol^–^^1^) at the Minimum and Selected Atoms for the Three Anions Used in This Work

anion	*V*_s,min_	*V*_s,Au_	*V*_s,I_^a^	*V*_s,I_^b^
[AuI_4_]^−^	–93.5	–74.0	–60.0	–
[AuI_2_]^−^	–97.3	–92.2	–77.2	–
[I_3_]^−^	–94.1	–	–73.9	–90.7

a At iodine’s σ-hole.

b At the belt of the central iodine atom in [I_3_]^−^.

The MEP values for the three anions are negative over the entire surface, as expected. In [AuI_4_]^−^, four equivalent global minima are found along the bisectors of the I–Au–I angles in the molecular plane (−93.5 kcal·mol^–1^, see Figure 3a). There are also four equivalent global maxima (least negative values), which are at the extensions of the four Au–I bonds (σ-holes, – 60.0 kcal·mol^–1^). The MEP is also less negative (compared to the minima) above and below the Au atom (π-holes, – 74.0 kcal·mol^–1^). The terms σ/π-hole have been used before to define regions with negative potential.^13,22^

The MEP surfaces of the linear anions are very similar, with two symmetrically equivalent belts where the MEP (see Figure 4b,c) is minimum. These belts are located in the middle of the Au–I bond in [AuI_2_]^−^ or I–I bond in [I_3_]^−^ with similar MEP values (−97.3 kcal·mol–1and −94.1 kcal·mol–1 for [AuI_2_]^−^, and [I_3_]^−^, respectively). There are also two equivalent maxima (least negative values), which are at the extensions of the Au–I or I–I bonds (σ-holes, – 77.2 kcal·mol–1 and −72.9 kcal·mol–1 for [AuI_2_]^−^, and [I_3_]^−^, respectively). From this analysis, it is clear that the Au···I short contacts observed in **1** and **2** do not originate from packing effects that pursue the least repulsive positioning of atoms, since the least repulsive combination would be the σ-hole···σ-hole interaction (I_3_Au^III^–I···I–Au^I^–I), instead of the Au···I contacts observed in both compounds.

**Figure 5:**
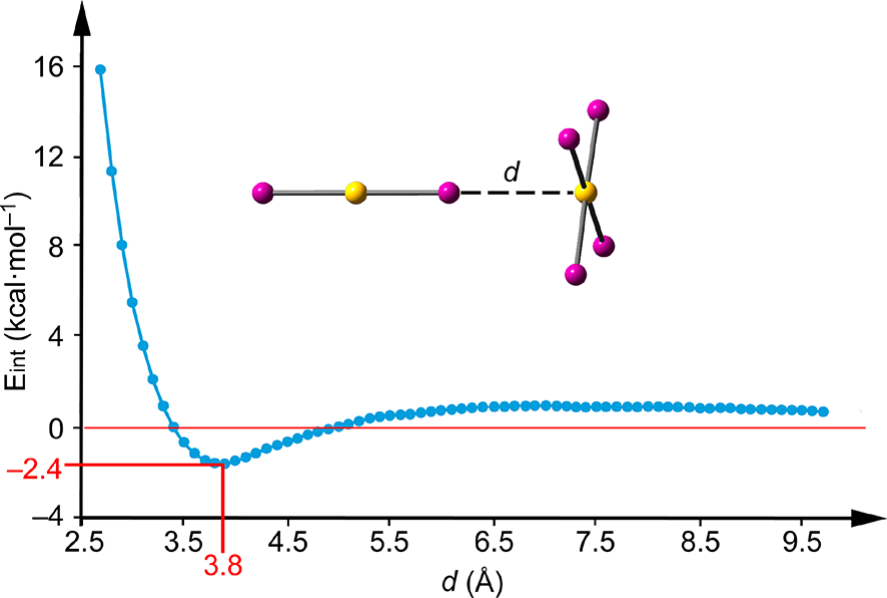
Energetic profile obtained by varying the interatomic distance (*d*) in the [AuI_4_]^−^···[AuI_2_]^−^ dimer at the PBE0-D3/def2-TZVP level of theory.

In order to further analyze the formation of the anion···anion [AuI_4_]^−^···[AuI_2_]^−^ complex, the energy profile (interaction energy vs distance, Figure 5) for the [AuI_4_]^−^···[AuI_2_]^−^ dimer was computed in the gas phase and in solution. The dianionic dimer is not stable in the gas phase where the monomers separate to infinitum. In the solid state of **1**, the anion···anion [AuI_4_]^−^···[AuI_2_]^−^ dimer is under the influence of the surrounding TMA molecules, which are obviously crucial for the stabilization of the anion···anion dimer. This effect has been modeled by computing the dimer using a continuum solvation model and the dielectric constant of water. The [AuI_4_]^−^···[AuI_2_]^−^ energy profile shows that the dimer is energetically favorable in water (Figure 5), as two separate monomers are less stable than the dimer by 2.4 kcal·mol^–1^ and the dissociation barrier is 2.9 kcal·mol^–1^. This result discloses that the electrostatic repulsion of the anion···anion dimer can be balanced by a convenient environment and that this interaction may exist in solution even without the presence of the counterion. While the dielectric constant of any crystalline compound is not known, it can be expected that the stabilization of the [AuI_4_]^−^···[AuI_2_]^−^ dimer is higher in the ionic environment of the crystalline salt than in solution. Similar results have been reported^23^ for the [I_3_]^−^···[I_3_]^−^ dimer, demonstrating its stabilization in different solvents.

**Figure 6:**
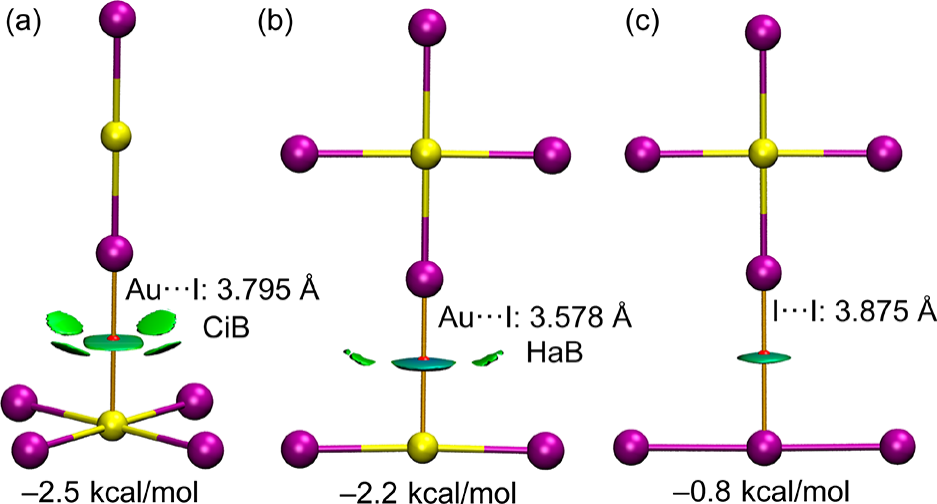
QTAIM distribution of intermolecular bond critical points (red spheres) and bond paths for the optimized π-hole (a) and σ-hole (b) dimers of [AuI_4_]^−^···[AuI_2_]^−^ and [AuI_4_]^−^···[I_3_]^−^ (c) in water solvent. The superimposed NCIplot isosurfaces (RDG isovalue = 0.45 au) is shown. The cutoff ρ = 0.04 au has been used. Color range −0.02 au ≤ (signλ_2_) ρ ≤ 0.02 au. Level of theory: PBE0-D3/def2-TZVPP.

The other binding mode between the [AuI_4_]^−^ and [AuI_2_]^−^ anions observed in the solid state of compound **2** (see Figure 3c), where one Au–I bond of [AuI_4_]^−^ is pointing to the central Au (or I) atom of the linear anion ([AuI_2_]^−^ or [I_3_]^−^), has been also optimized in water, finding a stable minimum. Both binding modes (dimers of **1** and **2**) are represented in Figure 6 along with the QTAIM/NCIplot analysis (see computational methods in SI for details). Both QTAIM and NCIPlot methods combined are very useful to reveal noncovalent interactions in real space. In all dimers, the interaction is characterized by a bond critical point (CP, small red sphere) and bond path (orange line) connecting the Au to the I or interconnecting both I atoms. The interactions are further characterized by green/bluish (sign(λ_2_)ρ < 0) RDG isosurfaces, thus revealing attractive interactions. This is confirmed by the interaction energies that are favorable for the three dimers in water, ranging from −0.8 kcal/mol for the I···I interaction in **2** to −2.5 kcal/mol for the I···Au(III) and I···I contacts in **1**. In the latter, the NCIplot analysis reveals the presence of four small isosurfaces between the I atoms of both anions that further contribute to the stabilization of the assembly. It is worth mentioning that the optimized distance of the I_4_Au(III)···I–Au(I)–I dimer (Figure 6a) is almost identical to that found in the solid state (3.794 Å), further suggesting that the isolated [AuI_2_]–···[AuI_4_]^−^ dimer found in compound **1** does not originate from packing effects. For the other binding mode observed in compound **2** (Figure 6b), the distance of the optimized dimer is significantly shorter than that in the solid state (>4 Å). This is likely due to the fact that compound **2** forms polymeric chains in the solid state instead of isolated dimers. Therefore, in the theoretical dimer, the Au···I interaction is overestimated with respect the experimental situation where the Au atom is establishing two concurrent Au(I)···I contacts.

**Figure 7:**
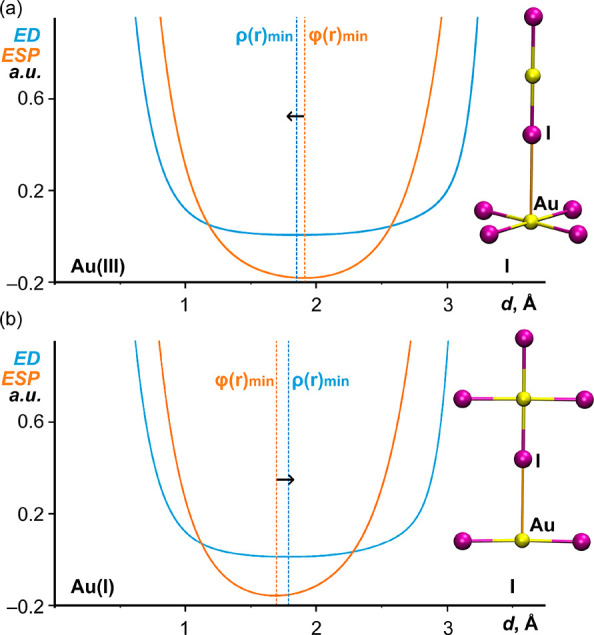
ED (blue curves) and ESP (orange curves) 1D profiles along the Au(III)···I (a) and Au(I)···I bond paths for the [AuI_4_]^−^···[AuI_2_]^−^ dimers.

In order to shed light into the nature of the Au···I contacts in the dimers shown in Figure 6 (coinage vs halogen bonds), analysis of the order of electron density (ED; ρ(r)min) and electrostatic potential (ESP; φ(r)min) minima has been carried out in their 1D profiles along the Au(III)···I and Au(I)···I bond paths (these paths are shown in orange in Figure 6a,b, respectively, and also in Figure 7). The QTAIM methodology is based on the concept of atomic basins that are assigned using the zero-flux condition [∇ρ(*r*)·*n*(*r*) = 0]^24^ in the electron density, and the corresponding surfaces are used to determine the interatomic boundaries. Similar boundaries can be also determined using the electrostatic potential, namely ∇φ(*r*)·*n*(*r*) = 0,^25^ defining the bonded electroneutral centers.^25−28^ The resulting difference in the interatomic boundaries of Au···I atoms is useful to reveal which of them is acting as Lewis base and which of them as Lewis acid. That is, in any donor–acceptor interaction, the φ(*r*)_min_ is shifted toward the nucleophilic atom, while the ρ(*r*)_min_ is shifted toward the electrophilic one.^29^

Figure 7 shows the 1D profiles of the ED and ESP functions along the Au(III)···I and Au(I)···I bond paths. Interestingly, an opposite behavior for both dimers is observed. That is, in the Au(III)···I dimer, the iodine atom is acting as nucleophile and the Au(III) atom as an electrophile since an evident shift of the ED minima toward the Au(III) electron density basin is appreciated (see small arrow in Figure 7a). This is evidence that the I atom is partially donating electrons to the electrophilic π-hole of Au(III) metal center. This result is in line with the MEP analysis that shows less negative MEP at the Au(III) atom than at the I atom of the [AuI_2_]^−^ anion. Therefore, this interaction can be termed as CiB, in line with recent investigations of [AuCl_4_]^−^ and [AuBr_4_]^−^ compounds.^13,14^ In contrast, in the Au(I)···I dimer, the Au(I) atom is acting as nucleophile and the iodine atom as an electrophile since a significant shift of the ED minima to the iodine electron density basin is observed. This is evidence that, in this case, the d^10^-Au(I) atom is partially donating electrons to the electrophilic σ-hole of iodine. This also agrees well with the MEP analysis that shows a large σ-hole at the extension of the Au(III)–I bonds. Consequently, the I···Au(I) interaction can be defined as a halogen bond. Recent investigations have demonstrated the nucleophilic nature of Au(I) complexes in halogen bonded assemblies.^30^

## Conclusion

In conclusion, experimental evidence is given that the gold atoms of [AuI_4_]^−^ anions form short contacts with the anionic [AuI_2_]^−^ nucleophiles in the solid state. Computations show that in **1**, where only Au(III)···I contacts are found, this interaction is attractive, and their orthogonal directionality is consistent with π-hole CiBs in line with other anion···anion interactions enabled by CiBs that have been rationalized by anisotropic distribution of the electron density in the anions.^13^ Interestingly, the Au(I)···I contacts in compound **2**, responsible for the formation of 1D supramolecular assemblies, can be defined as halogen bonds, since the Au(I) is acting as nucleophile. The isolated dimer observed in **1**, flanked by 12 TMA cations, is unprecedented in the literature. Moreover, density functional theory calculations suggest that the CiB driven anion···anion interaction that holds together the [AuI_4_]^−^·and·[AuI_2_]^−^ anions may exist also in solution at high concentrations.

This investigation is expected to attract the attention of not only researchers working in the fields of crystal engineering, supramolecular chemistry, and theoreticians but also those working in material science, since these anion···anion CiB and HaB interactions might be useful for the design and control of structural features of materials like, for example, perovskites for solar cells.

## Supporting Information Available

Additional crystallographic data, synthesis, FTIR-ATR/Raman data, and computational details along with further discussion (PDF)
