# Supplementary material for: Anion···Anion [AuI4]−···[AuI2]− Complex Trapped in the Solid State by Tetramethylammonium Cations
Source: Cryst Growth Des. 2022 Sep 28;22(11):6539–44. doi: 10.1021/acs.cgd.2c00749 (PMC9635596; doi:10.1021/acs.cgd.2c00749)
Supplement: Supplementary file 1 — cg2c00749_si_001.pdf [file cg2c00749_si_001.pdf]

Electronic Supplementary information

for manuscript

Anion···anion $[AuI_4]^-$···$[AuI_2]^-$ complex trapped in the solid state by tetramethylammonium cations

Luca Andreo[a], *Rosa M. Gomila*[b], *Emanuele Priola**[a], *Alessia Giordana*[a], *Stefano Pantaleone*[a], *Eliano Diana*[a], *Ghodrat Mahmoudi**[c], *and Antonio Frontera**[c]

[a] Department of Chemistry, Università degli Studi di Torino, Via Pietro Giuria 7, 10125 Torino (Italy)

[b] Department of Chemistry, Universitat de les Illes Balears, Crta. de Valldemossa km 7.5, 07122 Palma de Mallorca (Baleares) (Spain

[c] Department of chemistry, Faculty of Science, University of Maragheh, Maragheh (Iran)

[*] Corresponding Authors: Emanuele Priola: emanuele.priola@unito.it, Ghodrat Mahmoudi: ghodratmahmoudi@gmail.com, Antonio Frontera: toni.frontera@uib.es.

Materials and Methods

Gold(I) iodide (AuI, 99.9% trace metal basis), tetramethylammonium iodide ($(CH_3)_4NI$), 99%) and hydroiodic acid (HI, 57 wt.% in H_2O, distilled, stabilized, 99.95%) were purchased from Sigma Aldrich. Gold(III) hydroxide ($Au(OH)_3$, Au 79% min) was purchased from Alfa Aesar. Ethanol (96% vol.) was purchased from VWR Chemicals. All reagents and solvent were used as received.

Synthesis

$(Me_4N)_2(AuI_2)(AuI_4)$ (**1**): AuI (100 mg, 0.3 mmol) and Me_4NI (60 mg, 0.3 mmol) were dissolved in ethanol (20 ml). The resulting yellow solution was heated to reflux for 4 hours under constant stirring. The mixture was then allowed to cool, filtered, and allowed to slowly evaporate. After 5 days, dark red prismatic crystals of compound **1** were filtered, washed with cold ethanol and dried in air. Yield: 38 mg, 39%. ATR-FTIR (cm^{-1}): 3012 m, 2962 m, 2905 w, 1473 s, 1440 m, 1412 sh, 1402 m, 1394 m, 1285 w, 1260 s, 1094 m, 1018 s, 943 vs, 916 sh, 862 w, 795 m. FT-Raman (cm^{-1}): 157 vw, 143 vs, 125 s, 109 vw.
The proposed stoichiometry for the reaction is the following:

$$2\ (CH_3)_4NI + 4\ AuI \rightarrow [(CH_3)_4N]_2(AuI_2)(AuI_4) + 2\ Au$$

$(Me_4N)(AuI_2)_{0.5}(AuI_4)(I_3)_{0.5}$ (**2**): $Au(OH)_3$ (100 mg, 0.4 mmol) and Me_4NI (80 mg, 0.4 mmol) were put in ethanol (20 ml). Under constant stirring, 0.16 ml (1.2 mmol) of HI were slowly added to dissolve $Au(OH)_3$; the mixture was heated to reflux for 4 hours under constant stirring. The resulting solution was then allowed to cool, filtered and allowed to slowly evaporate. After 5 days, dark red prismatic crystals of compound **2** were filtered, washed with cold ethanol and dried in air. Yield: 122 mg, 48%. ATR-FTIR (cm^{-1}): 3018 w, 2978 vw, 1474 vs, 1440 s, 1428 s, 1406 m, 1372 m, 1285 w, 1220 vw, 1164 m,br, 1063 m, 1043 m, 1035 sh, 1001 w, 945 s, 874 w, 802 w. FT-Raman (cm^{-1}): 154 sh, 142 s, 125 s, 109 m.
The proposed stoichiometry for the reaction is the following:

$$4\ (CH_3)_4NI + 4\ Au(OH)_3 +\ 14\ HI \rightarrow 2\ [(CH_3)_4N]_2(AuI_2)_{0.5}(I_3)_{0.5}(AuI_4) + 12\ H_2O + I_3\ +\ [AuI_2]\ + 2\ H^+$$

Detail of experimental procedures

Single crystal data of compound **1**-**2** have been collected on a Gemini R Ultra diffractometer (Agilent Technologies UK Ltd., Oxford, U.K.) using graphite-monochromatic Mo Kα radiation (λ = 0.71073 Å) with the ω-scan method. CrysAlisPro software has been used for retrieving cell parameters, for performing data reduction and for absorption correction (with multi-scan technique). For the crystal of **2**, a merohedral twinning with twinning matrix (0 1 0 -1 0 0 0 0 1) have been detected and the intensities corrected. All structures were solved by direct methods using ShelXS-14[1] and refined with full-matrix least-squares on F^2 using the SHELXL-14[1] using Olex^2 program.[2] All non-hydrogen atoms have been anisotropically refined. Hydrogen atoms have been calculated and riding on the corresponding atom.

Structures images have been obtained using Mercury. The crystallographic data for **1**-**2** have been deposited in Cambridge Crystallographic Data Centre as supplementary publications under the CCDC numbers 2170930-

2170931. This information can be obtained free of charge from the Cambridge Crystallographic Data Centre via www.ccdc.cam.ac.uk/data_request/cifcodeCCDC.

Attenuated Total Reflectance (ATR-FTIR) spectra were recorded using a Bruker Vertex 70 spectrophotometer, equipped with a Harrick MVP2 ATR cell and DTGS detector. FT-Raman spectra were obtained with the same instrument, equipped with the RAMII accessory, by exciting with a 1064 nm laser. The adopted resolution was equal to 4 cm^{-1} in all cases.

Crystallographic Tables

Table 1. Crystal data and structure refinement for $(Me_4N)_2(AuI_2)(AuI_4)$ (**1**).

Empirical formula	$C_8H_{24}Au_2I_6N_2$
Formula weight	1303.63
Temperature/K	297
Crystal system	Orthorhombic
Space group	*Cmcm*
a/Å	12.9498(5)
b/Å	14.7357(8)
c/Å	13.1896(7)
α/°	90
β/°	90
γ/°	90
Volume/Å^3	2516.9(2)
Z	4
ρ_{calc}g/cm^3	3.440
μ/mm^{-1}	79.317
F(000)	2248.0
Crystal size/mm^3	0.15 × 0.15 × 0.12
Radiation	CuKα (λ = 1.54184)
2Θ range for data collection/°	9.092 to 135.846
Index ranges	$-15 \le h \le 10, -17 \le k \le 12, -15 \le l \le 10$
Reflections collected	11802
Independent reflections	1254 [R_{int} = 0.0699, R_{sigma} = 0.0190]
Data/restraints/parameters	1254/0/54
Goodness-of-fit on F^2	1.112
Final R indexes [I>=2σ (I)]	R_1 = 0.0284, wR_2 = 0.0729
Final R indexes [all data]	R_1 = 0.0299, wR_2 = 0.0739
Largest diff. peak/hole / e Å^{-3}	0.92/-1.29

Table 2. Bond Lengths for $(Me_4N)_2(AuI_2)(AuI_4)$ (**1**).

**Atom**	**Atom**	**Length/Å**		**Atom**	**Atom**	**Length/Å**
Au1	I1^1	2.6353(18)		Au2	I3	2.560(3)
Au1	I1	2.6354(18)		N1	C2^3	1.473(19)
Au1	I2^2	2.6372(17)		N1	C2	1.473(19)
Au1	I2	2.6372(17)		N1	C1^3	1.48(2)
Au2	I4	2.538(3)		N1	C1	1.48(2)

^{1}=+X,+Y,1/2-Z; ^{2}=-X,+Y,+Z; ^{3}=+X,1-Y,1-Z

Table 3. Bond Angles for $(Me_4N)_2(AuI_2)(AuI_4)$ (**1**).

Atom	Atom	Atom	Angle/°		Atom	Atom	Atom	Angle/°
$I1^1$	Au1	I1	175.27(9)		$C2^3$	N1	C2	111(2)
$I1^1$	Au1	I2	90.080(2)		$C2^3$	N1	C1	111.3(12)
I1	Au1	I2	90.080(2)		C2	N1	C1	108.3(12)
$I1^1$	Au1	$I2^2$	90.080(2)		$C2^3$	N1	$C1^3$	108.3(12)
I1	Au1	$I2^2$	90.080(2)		C2	N1	$C1^3$	111.3(12)
$I2^2$	Au1	I2	176.12(9)		$C1^3$	N1	C1	107(2)
I4	Au2	I3	180.0					
^{1}=+X,+Y,1/2-Z; ^{2}=-X,+Y,+Z; ^{3}=+X,1-Y,1-Z								

Table 4. Crystal data and structure refinement for $(Me_4N)(AuI_2)_{0.5}(AuI_4)(I_3)_{0.5}$ (**2**).

Empirical formula	$C_4H_{12}Au_{0.75}I_{3.25}N$
Formula weight	634.30
Temperature/K	293(2)
Crystal system	Tetragonal
Space group	P-42_1m
a/Å	9.7341(13)
b/Å	9.7341(13)
c/Å	13.442(3)
α/°	90
β/°	90
γ/°	90
Volume/Å^3	1273.7(4)
Z	4
ρ_{calc}g/cm^3	3.308
μ/mm^{-1}	16.509
F(000)	1098.0
Crystal size/mm^3	0.9 × 0.12 × 0.11
Radiation	MoKα (λ = 0.71073)
2Θ range for data collection/°	7.368 to 52.736
Index ranges	$-7 \le h \le 11, -12 \le k \le 9, -12 \le l \le 16$
Reflections collected	3686
Independent reflections	1362 [R_{int} = 0.0519, R_{sigma} = 0.0557]
Data/restraints/parameters	1362/0/54
Goodness-of-fit on F^2	1.057
Final R indexes [I>=2σ (I)]	R_1 = 0.0734, wR_2 = 0.1744
Final R indexes [all data]	R_1 = 0.1174, wR_2 = 0.2007
Largest diff. peak/hole / e Å^{-3}	2.58/-1.04
Flack parameter	0.029(18)

Table 5. Bond Lengths for $(Me_4N)(AuI_2)_{0.5}(AuI_4)(I_3)_{0.5}$ (**2**).

Atom	Atom	Length/Å		Atom	Atom	Length/Å
Au1	I3	2.635(5)		N1	$C1^3$	1.42(6)
Au1	I1	2.624(5)		N1	$C1^4$	1.42(6)
Au1	I2	2.628(3)		N1	$C1^5$	1.42(6)
Au1	$I2^1$	2.628(3)		N2	C2	1.45(5)
Au2	I4	2.784(6)		N2	$C2^6$	1.45(5)
Au2	$I4^2$	2.784(6)		N2	$C2^7$	1.45(5)
I4	I5	2.784(6)		N2	$C2^8$	1.45(5)
N1	C1	1.42(6)				

^{1}=1-X,-Y,+Z; ^{2}=-X,1-Y,+Z; ^{3}=+Y,-X,1-Z; ^{4}=-Y,+X,1-Z; ^{5}=-X,-Y,+Z; ^{6}=1-Y,-1+X,-Z; ^{7}2-X,-Y,+Z; ^{8}1+Y,1-X,-Z

Table 6. Bond Angles for $(Me_4N)(AuI_2)_{0.5}(AuI_4)(I_3)_{0.5}$ (**2**).

Atom	Atom	Atom	Angle/°		Atom	Atom	Atom	Angle/°
I1	Au1	I3	180.0		C1^{3}	N1	C1^{4}	107(3)
I1	Au1	I2	89.46(10)		C1^{4}	N1	C1	114(5)
I1	Au1	I2^{1}	89.46(10)		C1^{5}	N1	C1	107(3)
I2	Au1	I3	90.54(10)		C2	N2	C2^{6}	110(2)
I2^{1}	Au1	I3	90.54(10)		C2	N2	C2^{7}	109(4)
I2	Au1	I2^{1}	178.9(2)		C2^{6}	N2	C2^{8}	109(4)
I4^{2}	Au2	I4	177.8(3)		C2	N2	C2^{8}	110(2)
C1^{3}	N1	C1	107(3)		C2^{6}	N2	C2^{7}	110(2)
C1^{4}	N1	C1^{5}	107(3)		C2^{7}	N2	C2^{8}	110(2)
C1^{3}	N1	C1^{5}	114(5)		I4^{2}	I5	I4	177.8(3)
^{1}=1-X,-Y,+Z; ^{2}=-X,1-Y,+Z; ^{3}=-Y,+X,1-Z; ^{4}=-X,-Y,+Z; ^{5}=+Y,-X,1-Z; ^{6}=1-Y,-1+X,-Z; ^{7}=2-X,-Y,+Z; ^{8}=1+Y,1-X,-Z								

Figure S1:

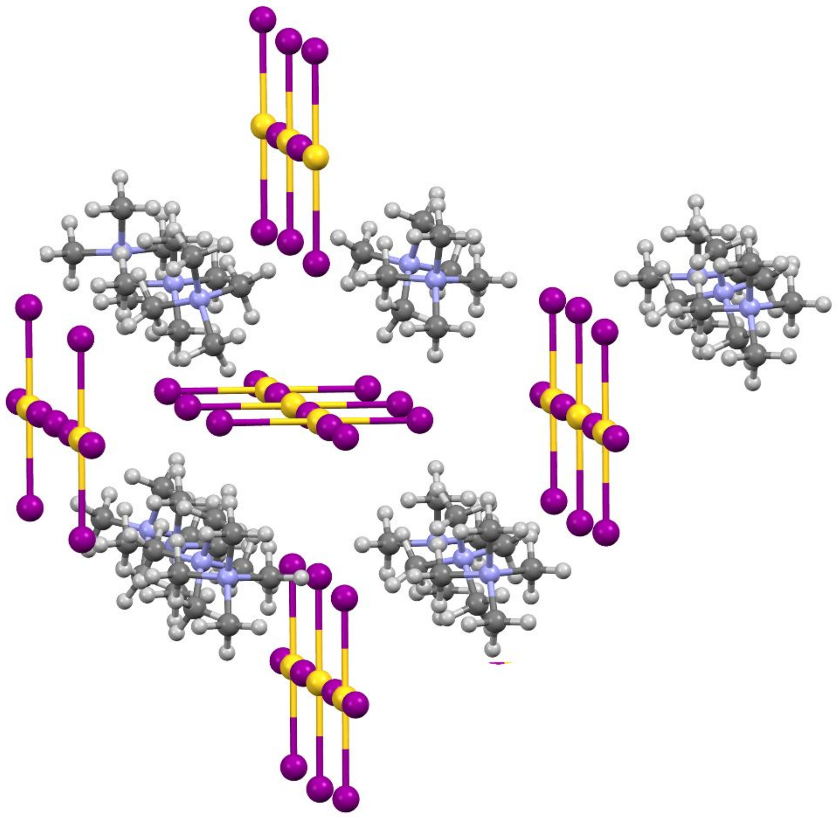

Figure S1. X-ray packing of compound **2** showing the counter-ions (TMA)

Theoretical methods

The energetic and geometric features of the adducts analyzed in this work were calculated at the PBE0[3]-D3[4]/def2-TZVPP[5] level of theory. The geometries were fully optimized using the crystallographic coordinates as starting points. For gold and iodine, the inner shell electrons are modelled by ECPs (ECP-60 scheme),[6] which also accounts for scalar relativistic effects. The TURBOMOLE 7.0 program has been used for the energetic calculations[7] and the conductor-like solvation model (COSMO) to account for solvent effects.[8] Molecular electrostatic potential (MEP) surfaces have been computed at the same level of theory and represented using the 0.001 a.u. isovalue of electron density to map the electrostatic potential. The QTAIM[9] and NCIPlot[10,11] analyses have been performed using the multiwfn program[12] at the same level of theory and represented using VMD software.[13]

References

[1] Sheldrick, G. M. *Acta Cryst.* **2015**, *C71*, 3-8.

[2] Dolomanov, O. V.; Bourhis, L. J.; Gildea, R. J.; Howard, J. A. K.; Puschmann, H. *J. Appl. Cryst.* **2009**, *42*, 339-341

[3] Adamo, C.; Barone, V. *J. Chem. Phys.* **1999**, *110*, 6158−6170.

[4] Grimme, S.; Antony, J.; Ehrlich, S.; Krieg, H. *J. Chem. Phys.* **2010**, *132*, 154104.

[5] Weigend, F. *Phys. Chem. Chem. Phys.* **2006**, *8*, 1057−1065.

[6] Andrae, D.; Haeussermann, U.; Dolg, M.; Stoll H.; Preuss, H. *Theor. Chim. Acta* **1990**, *77*, 123−141.

[7] Ahlrichs, R.; Bär, M.; Häser, M.; Hom, H.; Kölmel, C. *Chem. Phys. Lett*. **1989**, *162*, 165–169.

[8] Schäfer, A.; Klamt, A.; Sattel, D.; Lohrenz, J. C. W.; Eckert, F .*Phys. Chem. Chem. Phys*. **2000**, *2*, 2187–2193

[9] Bader, R. F. W. *Chem. Rev.* **1991**, *91*, 893−928.

[10] Johnson, E. R.; Keinan, S.; Mori-Sánchez, P.; Contreras-García, J.; Cohen, A. J.; Yang, W. *J. Am. Chem. Soc.* **2010**, *132*, 6498-6506.

[11] Contreras-García, J.; Johnson, E. R.; Keinan, S.; Chaudret, R.; Piquemal, J.-P.; Beratan, D. N.; Yang, W. *J. Chem. Theory Comput.* **2011**, *7*, 625-632.

[12] Lu, T.; Chen, F. *J. Comput. Chem*. **2012**, *33*, 580-592.

[13] Humphrey, W.; Dalke, A.; Schulten, K. *J. Molec. Graph.*, **1996**, 33-38
